# How do STEM graduate students perceive science communication? Understanding science communication perceptions of future scientists

**DOI:** 10.1371/journal.pone.0274840

**Published:** 2022-10-03

**Authors:** Tessy S. Ritchie, Dione L. Rossiter, Hannah Bruce Opris, Idarabasi Evangel Akpan, Simone Oliphant, Melissa McCartney

**Affiliations:** 1 Department of Chemistry & Life Science, United States Military Academy, West Point, NY, United States of America; 2 Science at Cal, University of California, Berkeley, Berkeley, CA, United States of America; 3 STEM Transformation Institute, Florida International University, Miami, FL, United States of America; 4 Department of Biological Sciences, Florida International University, Miami, FL, United States of America; Lund University: Lunds Universitet, SWEDEN

## Abstract

Increasingly, communicating science to the public is recognized as the responsibility of professional scientists; however, these skills are not always included in graduate training. In addition, most research on science communication training during graduate school, which is limited, has been program evaluation or literature reviews and does not report on or seek to understand graduate student perspectives. This research study provides a comprehensive analysis of graduate-level science communication training from the perspective of STEM graduate students. Using a mixed-methods approach, this study aimed to investigate where graduate students are receiving science communication training (if at all), what this training looks like from the student’s point of view, and, for graduate students that are engaging in science communication, what do these experiences look like. This study also explores how graduate students define science communication. Taken together, these results will give graduate students a voice in the development of science communication trainings and will remove barriers and increase equity in science communication training.

## Introduction

Science communication has taken on many definitions. Communicating science can be divided into scientific communication, which refers to scientists sharing their work inside their community, and science communication, which refers to sharing science with non-experts [1]. Both aspects of communicating science are important, and both are integral parts of a being a scientist. Whether it be giving an oral presentation at a scientific conference or a public talk at a science center, writing a scientific journal article or an op-ed, or simply engaging in a conversation with a colleague or a friend, scientists are communicating.

When scientists are able to effectively communicate with each other, society gets better science. When scientists are able to effectively communicate with the public, the general public gets a wealth of benefits, including an increase in public support for science and science funding, more informed science policy at all levels, clearer guidelines for environmental and public health initiatives, and, perhaps most importantly, citizens that practice evidence-based decision making; and thus, better informed voters. Inclusive and strategic science communication initiatives can even help eliminate societal structures that perpetuate inequality (i.e. pay gaps and knowledge gaps) and provide role models to repair “leaky pipelines” for women and members of traditionally underrepresented communities.

The ability to communicate effectively is an important skill for scientists regardless of career path, whether that be in academia, industry, or careers away from the bench (including, but not limited to, nonprofit, government, business, law, informal and formal K-12 education, etc.). In an academic setting, science communication is expected between university scientists and undergraduate, graduate, and postdoctoral scholars in classrooms and labs. For both academic and industry scientists, communication between colleagues is required to foster innovation and collaboration, to share scientific results, and to encourage scientific discourse and critique. Examples of science communication include scientific publications, funding proposals, and conference oral or poster presentations. Both groups have an obligation to, and are sometimes required to, communicate to stakeholders like community members, board members or, in the case of any publicly funded research, the taxpayer. Scientists who choose careers away from the bench are often faced with the additional challenge of communicating with nonscientific audiences after years of technical training and learning how to communicate and engage in an academic setting, with little or no training in communicating science to non-academic audiences.

A recent study of descriptive analysis of 142,000 job advertisements found that oral communication and written communication are the top two in demand 21st-century skills, expressed as a proportion of total job advertisements examined [2]. The study further showed that these skills are important for workplace success but are scarce in the applicant pool. More specific to STEM, a recent study of U.S.-trained doctoral chemists from academia, industry, and government were interviewed about the activities they conduct on a day-to-day basis and the knowledge and skills required to successfully complete these activities. Communication skills were the second most often mentioned skill by chemists in the interviews [3]. A similar study, focusing on bachelors-leveled chemists, uncovered a disconnect between the skills cross-sector employers desire and those they expect from their new hires’ formal instruction. To address this disconnect, the study recommends including interprofessional skills, which include communication skills, into the scientific curriculum [4]. Similar results are found in the biomedical sciences, with communication skills being considered requisite for success in today’s economy [5]. Research also suggests that engaging in and receiving active mentoring in science communication play a significant role in a young scientist’s intent to pursue an academic career [6].

Graduate school curriculums are designed to provide comprehensive training, preparing scientists to be experts in their field. Once in the workforce, there are rarely any required continuing professional development requirements or opportunities. Therefore, graduate school is likely the final stage of traditional lecture and course-based learning. As noted previously, scientists are required to communicate their science to other scientists, to their students, to funding agencies, and to the public, yet no formal training in science communication is required. We argue that science communication training should be an integral component of graduate-level coursework.

Most research on science communication training during graduate school, which is limited, has been program evaluation or literature reviews [7–11]. Outside of workshop and/or conference reports, there is little evidence in the peer-reviewed literature on graduate students’ perspective of science communication training, and there are few peer-reviewed, empirical studies on how graduate students define, perceive, or engage in science communication. In December of 2013, COMPASS convened the #GradSciComm workshop, bringing together a select group of 30 science communication trainers, scholars, science society staff, funders, administrators, and graduate student leaders [11]. As part of this workshop, participants worked together to define the core concepts and essential knowledge and skills of science communication [11]. While this workshop made great gains in mapping the pathways to integrate science communication training into STEM graduate education, it was ultimately a self-selecting group that included established scientists, faculty, and professional science communicators, and did not provide direct evidence of how STEM graduate students themselves define and prioritize science communication. While we fully recognize that many STEM graduate students have indeed received excellent training either from within their institution or from external opportunities, we also recognize the absence of the graduate student perspective from the literature [9, 10, 12–20].

Our research is not meant to compare or evaluate existing training programs, rather it comes from an intent to give STEM graduate students more of a voice in the conversation. In this study, we aim to expand the existing knowledge base on how current STEM graduate students define science communication, practice and engage in science communication, and how they envision their future as science communication practitioners. Through quantitative and qualitative data analysis, feedback is presented from STEM graduate students themselves as a way to initiate new lines of inquiry around future development of science communication opportunities for STEM graduate students. As a mixed-methods phenomenological study, this research is not specifically seeking to test existing theories about how or why graduate students decide to engage in science communication [21, 22] nor is it meant to compare or evaluate existing training programs. Instead, we quantitatively and qualitatively analyzed the unique ways students experience the same phenomenon as a way to compile a comprehensive description of STEM graduate students’ “lived experiences.” To the best of our knowledge, this study represents one of the only data sets from graduate students themselves, in which graduate students provide their perceptions of science communication training experiences, how they define science communication, how they have engaged in the process of science communication, and how they envision the future of science communication training.

## Methods

### Development of the Graduate Student Science Communication (GSSC) questionnaire

We identified key definitions and research questions provided in policy documents, research studies, editorials, and commentaries on the state of science communication and reviewed the literature describing specific communication skills. Using the information found, questions falling into four major categories were developed: 1) what previous science communication training (if any) have the participants experienced; 2) how do STEM graduate students define science communication; 3) what ways have the participants engaged in the process of science communication; and 4) how do the participants envision the future of science communication training.

### Modification of the GSSC questionnaire using a STEM graduate student focus group

A total of twelve graduate students from varied STEM disciplines were recruited to participate in a focus group at Florida International University (FIU), a R1 University in the southeastern United States. Each student was given an online link to the GSSC questionnaire and asked to answer to the best of their ability. Directly after the GSSC questionnaires were completed, the students participated in a focus group conducted by two researchers (S.O. and M.M.). Students were asked for feedback in regards to the wording of each question, the purpose of each question, the length of the entire questionnaire, and their overall experience while completing the questionnaire. The questionnaire was edited based on the feedback of the focus group, and questions that were identified as poorly written, confusing, too time consuming, or repetitive were removed. No individual data from focus group participants were included in the current analysis. The questionnaire was submitted to the Institutional Review Board at FIU and U.S. Naval Academy (USNA) and was granted IRB approval (FIU IRB # 106915) and (USNA.2018.0059-IR-EP7-A). Participant consent was collected via the consent form in the questionnaire. Participants marked the "I agree to participate in this research study" which allowed them to proceed to the rest of the questionnaire. Anyone who did not agree was not allowed to proceed with the questionnaire.

### Distribution of the GSSC questionnaire

The GSSC questionnaire was administered through Qualtrics (online survey software; Provo, Utah and Seattle, Washington). Links to the GSSC questionnaire and a description of the research study were sent to relevant listservs and distributed using social media. Two-hundred and nine graduate admissions offices and individual departments were contacted via email. The email provided information on the questionnaire and encouraged their students participate. The GSSC questionnaire was open from September 2018 through February 2019. A total of 273 responses were recorded, with 161 determined to be complete enough to include in the analysis (59% of total responses were analyzed). Questionnaires were completely anonymous and no incentives were given for completion of the questionnaire.

### Qualitative data analysis and inductive coding

Short answer response data were analyzed using inductive coding, a subset of thematic analysis [23], and Nvivo software (NVivo version 11.4, QSR International). Per definition, inductive coding is free from theoretical frameworks. Instead, inductive coding is completely driven by the participants’ responses [23]. Four researchers (T.S.R., H.B.O., I.A., and M.M.) read all of the short answer responses and independently created lists of the different perceptions, attitudes, and opinions that arose from participant responses. Initial findings were discussed among the four researchers and a preliminary code book was developed consisting of short, descriptive phrases that could be used to describe particular perceptions, attitudes, or opinions expressed by participants. Each short answer question was independently coded by two researchers. The pair of researchers then convened to discuss, further define, and reduce codes that were unclear. Analysis of coding considered only the presence or absence of specific themes within each short answer, not the frequency with which a single participant expressed a particular theme. Responses corresponding to more than one theme were coded to each code they corresponded with. Kappa values measuring inter-rater reliability (the extent to which researchers assign the same code to the same data) were over 0.8, which represent higher standards than recommended (0.65) [24]. The results of inductive coding analysis are presented in the results section using a series of Tables containing 3 columns (Tables 1–6, 8, 9). The first column contains the results from thematic analysis: the emerging code and the percentage of participants whose responses were determined to fall within each emerging code. The second column provides a brief description of the emerging code. The third column contains a sample of participant responses, with the coded text bolded and underlined for ease of reading.

**Table 1:** Skills STEM graduate students report being needed to communicate science.

Code	Description of code	Example responses
Knowing your audience (73% of participant)	Participants describe skills such as being able to recognize the abilities and the knowledge level of their audience and being able to frame science in an interesting and engaging way.	“Science Communication is the art of sharing factual truth in a manner that can be understood. It requires **recognizing the audience’s abilities, knowledge, and skill level** to adequately share scientific truths in a manner that will be accepted and believed.” “Science communication is the exchange of scientific ideas, concepts, and findings in terms that are familiar to all parties involved. Such communication is most successfully facilitated by those who **consider their target audience**, distill their message into a digestible form, and present it with clear language and a logical flow. Properly communicated science should confer upon the audience an understanding of what the problem/lesson is, how it affects or is affected by them, and how they can use/share the information.”
Using clear language (22% of participants)	Participants describe using concise language, free from jargon, which can be understood by the general public.	“Being able to **describe your science and it’s importance to an average person in an easily understandable way**” “Science communication is communicating to the general public or broad audience about science in the best medium (video, podcasts, social media) for these communities. **Typically there is less jargon** and the science is packaged for optimal understanding for people outside if the field.”
Knowing the science (6% of participants)	Participants describe the importance of knowing and understanding the science you are communicating.	“**Ability to understand technical information in a specific area of science** and ability to translate this information to people outside of the field” “Science communication is bringing back home **all of the knowledge we’ve learned from the field, in conferences, during our studies and from our research**. We tell our friends, families and colleagues around the world what we’re doing and what others have done to make life better for humanity.”
Establishing relationships (4% of participants)	Participants describe being able to establish and cultivate relationships between scientists and the public.	“**Establishing and cultivating relationships between scientists and broad, public audiences** through discussions, activities, resources, or other forms of engagement.” “The use of appropriate means (whatever you consider the best one for the specific context) to **create bridges between society and science****”**
No skills mentioned (2% of participants)	Participants do not mention any skills or they mention skills in such general terms that it is not possible to describe in specific terms.	“There are two main forms of science communication: from expert to public, and from expert to expert.” “An underestimated discipline, that requires many different skills and a very strong personal commitment.”

**Table 2:** Intended audiences STEM graduate students expect to engage with.

Code	Description of code	Example responses
General Public (expert to non-expert) (50% of participants)	Participants describe engaging with the general public.	“Science communication is the process of sharing factual information in such a way that a **general audience** can understand it but also appreciate it. You try to make the **average person** realise how awesome science already is by framing it in a way that is more appealing.” “Science communication is the dissemination of information related to complex science concepts, theories, or experiments that can be understood by **non-experts and the general public**. It also aims to describe this information in an engaging way.”
Other scientists (expert to expert) (2% of participants)	Participants describe ONLY engaging with other scientists, with no mention of the general public.	“science communication is the communication of science ideas, results, or implications, usually through **papers published in journals or spoken through conference presentations**" “A presentation of my research work in a **conference meeting** or just **informal talk with professors or other students**. But the main key is it is about the research in the lab.”
Both the general public and other scientists (34% of participants)	Participants describe engaging with the general public and with other scientists.	"Facilitating discussion around science-related issues by communicating the scientific, social, political, and personal aspects of the **issues** **across expert and non-expert groups** and media platforms." "Science communication is the process of disseminating scientific **information within the scientific community and outside of it**. It may consist of publications, seminars, podcasts, or any form of media."
No audience was mentioned (18% of participants)	Participants do not mention an audience or they mention audience in such general terms that it not possible to determine expert or non-expert	“The ability to explain ones research **to a large audience** making it as accessible and interesting as possible” “explaining and discussing scientific findings and experiments”

**Table 3:** Mediums through which STEM graduate students envision science communication. As described in the methods section, responses corresponding to more than one code were coded to each code they correspond with within the same question.

Code	Description of code	Example responses
Oral communication (15% of participants)	Participants describe oral communication including public lectures/talks, conversations, and interviews.	"Any attempt (from an expert or non-expert) to make scientific concepts understandable and communicate them via channels such as **public lectures**, writing, videos, or even **a face-to-face conversation**." "Science communication is the process of sharing information obtained from scientific experiments to your peers and to the general public through various forms of media, including but not limited to **oral** presentations, videos, posters, etc."
Written communication (13% of participants)	Participants describe written communication broadly or types of written communication including books, newspapers, and research publications.	“Science communication involves the effective sharing of scientific information or research results to colleagues and the general public, **through writing**, oral presentations, or illustrations, i.e., figures or diagrams.” “Dialogue about scientific ideas and topics through oral or **written communications**”
Social media (6% of participants)	Participants describe social media broadly or types of social media including YouTube, Twitter, Facebook, and Blogs.	“Science communication is communicating to the general public or broad audience about science in the best medium (video, podcasts, **social media**) for these communities. Typically there is less jargon and the science is packaged for optimal understanding for people outside if the field.” “Science communication is any avenue scientists use to disseminate their research. This can be through posters, talks, publications, interviews, podcasts, **tweets**, or media appearances.”
No medium mentioned (76% of participants)	Participants do not mention a medium or they mention a medium in such general terms that it is not possible to define any further	“Facilitating discussion around science-related issues by communicating the scientific, social, political, and personal aspects of the issues across expert and non-expert groups and **media platforms**.” “Science communication is **any avenue** scientists use to disseminate their research.”

**Table 4:** The purpose of science communication as perceived by STEM graduate students.

Code	Description of code	Example responses
Sharing science with a general audience (78% of participants)	Participants describe different ways of sharing science with a general audience, including increasing appreciation of science, increasing interest in science, and inspiring confidence in science.	“Translating scientific information into the language more commonly used by the general public in order to bridge the gap between academic knowledge and implementation of results in the community and **inspire widespread confidence and interest in science.**” “The effort of making complex ideas accessible in a simple way to non specialized audience. The effort of communicating the importance of a given research for the community. The effort of creating a rethorics which **makes the scientific enterprise more interesting**.”
Sharing science with other scientists (20% of participants)	Participants describe communication between scientists.	"Science communication is bringing your scientific research or knowledge to others in the world. **It may be in a more academic setting, where your audience mainly consists of other scientists**, or it could be for the general public, where people have widely variable science backgrounds." “Science communication is keeping **scientists outside your field** and the general public up to date and engaged with your research.”
Informing policy (16% of respondents)	Participants describe the importance of science communication as it relates to informing policy and informing voters.	“The ability to clearly articulate to the public and **policy makers** what you are doing and how it impacts them.” “Science communication is the capacity of evoking unfamiliar scientific concepts for the general public in a way that is familiar, simple, and understandable. Science communication can be a remarkable tool to create awareness among the non-scientific public about a particular topic. For example, **experts in science communication are genuinely needed in Capitol Hill to shape policymaking** related to relevant issues (e.g., global warming).”
Educating others about science (9% of participants)	Participants describe education others about the facts of science and to clarify misconceptions.	"Presenting science in any form (written, spoken, books) to a person not familiar with the field. Aimed to **educate about concepts and refute misconceptions**." “Distilling high-level information to those outside of one’s own specialized field. **Understanding where misconceptions lie**, and how the information being communicated ties into everyday life or real life scenarios.”
Encouraging the public to engage in science (4% of participants)	Participants describe a method to engage the public in science.	“Being able to tell the public about your research in an understandable and accessible way. Also **providing opportunities for the public to be involved in science**” “We science communicator try to make difficult science knowledge easier for the public to understand and try to **invite the public to care about and discuss science-related social issue**.”
No purpose mentioned (6% of participants)	Participants do not mention a purpose or they mention a purpose in such general terms that it is not possible to define any further	“simple nerd talk” “This is a rather vague question. Science and communication are both nouns, so taken as a singular concept, science communication is communication of scientific thought.”

**Table 5:** Participant responses to the question “what has stopped you from engaging in science communication related to a general science concept?” the resulting codes are shown in the left hand column, with a description of each code in the center column, and example responses from participants in the right hand column. Text from each response that is directly relevant to each code is in bold and underlined text.

Code	Description of code	Example responses
Unaware of opportunities (51% of respondents)	Participants describe being unaware of training opportunities and/or opportunities to engage in science communication.	“Opportunities at my university to present to the public **have been focused specifically on my field of study**” “**I was never made aware of opportunities to learn more about science communication** and the importance and benefits of science communication, such as networking opportunities, and the self-promotion as a PhD & researcher.”
Overstepping expertise (27% of respondents)	Participants describe feeling unqualified to speak on research outside of their area of expertise/field and not having enough knowledge to share.	"**I am not familiar enough with most of the general science concepts** beyond what is in the media to engage people in a knowledgeable science way." "**Not being an expert in the field** / not being considered qualified to educate others on general science concepts."
Nerves (14% of respondents)	Participants describe being afraid of the expectations of the public, being afraid to address controversial topics, and lack of confidence in general.	"**Afraid of the expectations set by the public** about who will communicate science to them." "I feel the ’general science concepts’ become scary because often people have their own unwavering opinions about the topics. **I prefer talking about things less controversial** to my audience than things that may be controversial or bring unhelpful criticism/tangents to the conversation."
Too busy (12% of respondents)	Participants describe a lack of time that is not connected to time in the lab.	"**I haven’t had time to volunteer** in the few opportunities that have been available." "**My time is currently very limited** and the extracurricular activities I have participated in have not focused on general science concept communication but more individualized activities."
Takes time away from the lab (7% of respondents)	Participants describe time away from the lab specifically, as well as pressure from PI to be in the lab and avoid distractions.	"I enjoy science communication very much so if I were to engage in it at the current time I would be harmfully distract myself from more immediate concerns, such as studying for comprehensive exams, and **more long term concerns such as maximizing time spent on research**." "Probably by workload and **pressure from my PI** that this kind of experience is not important. It is definitely a goal for grad school but it always gets put on the back burner for these reasons."
It is unnecessary (4% of respondents)	Participants describe a lack of interest in science communication.	"i think its **unnecessary**." "**No interest**—I think my own research (studying the mechanism of HIV infection) is far more interesting, and I have far more mastery of it"

**Table 6:** Participant responses to the question “what has stopped you from communicating your own thesis research to a non-scientific general audience?” the resulting codes are shown in the left hand column, with a description of each code in the center column, and example responses from participants in the right hand column. Text from each response that is directly relevant to each code is in bold and underlined text.

Code	Description of code	Example responses
Unaware of opportunities (35% of respondents)	Participants describe being unaware of training opportunities and/or opportunities to engage in science communication.	"**haven’t had the opportunity** or seen this kind of opportunity" "**I don’t have much opportunity** to do so (except in very informal occasions, like talking with my family or friends)."
Overstepping expertise (30% of respondents)	Participants describe feeling unqualified to speak on research outside of their area of expertise/field and not having enough knowledge to share.	"**Not far enough in research yet** to give a full presentation" "My research is **not yet at a point where I feel comfortable talking about it** to a non-scientific general audience."
It is unnecessary (13% of respondents)	Participants describe a lack of interest in science communication, think the public is not interested, or state that their research is not worth communicating.	“I feel it is so specific that **no one would understand or find it interesting,**” “It was technical and not applicable. **people would not care**.”
Thesis research is not transferrable to a general audience (11% of respondents)	Participants describe their thesis research as containing too much jargon, being too niche, not being relevant to public interests, and not being connected to the real world.	"My field is a very particular subset that **uses a lot of jargon, and I’m still trying to figure out how to translate that into language that can be understood** by the general public." "I’ve incorporated my research into public talks, **but it’s too niche so it’s never the center of the talk**."
Nerves (4% of respondents)	Participants describe being afraid of the expectations of the public, being afraid to address controversial topics, and lack of confidence in general.	"I feel very uncomfortable sharing my graduate research with non-**scientists because my research involves sacrificing birds. It’s not something I feel comfortable admitting in public spaces** due to the existence of groups like PETA." “language problems”
Too busy (4% of respondents)	Participants describe a lack of time that is not connected to time in the lab.	"I have **not had time**" "have **not had the time**"

### Descriptive statistics

**Fig 1:**
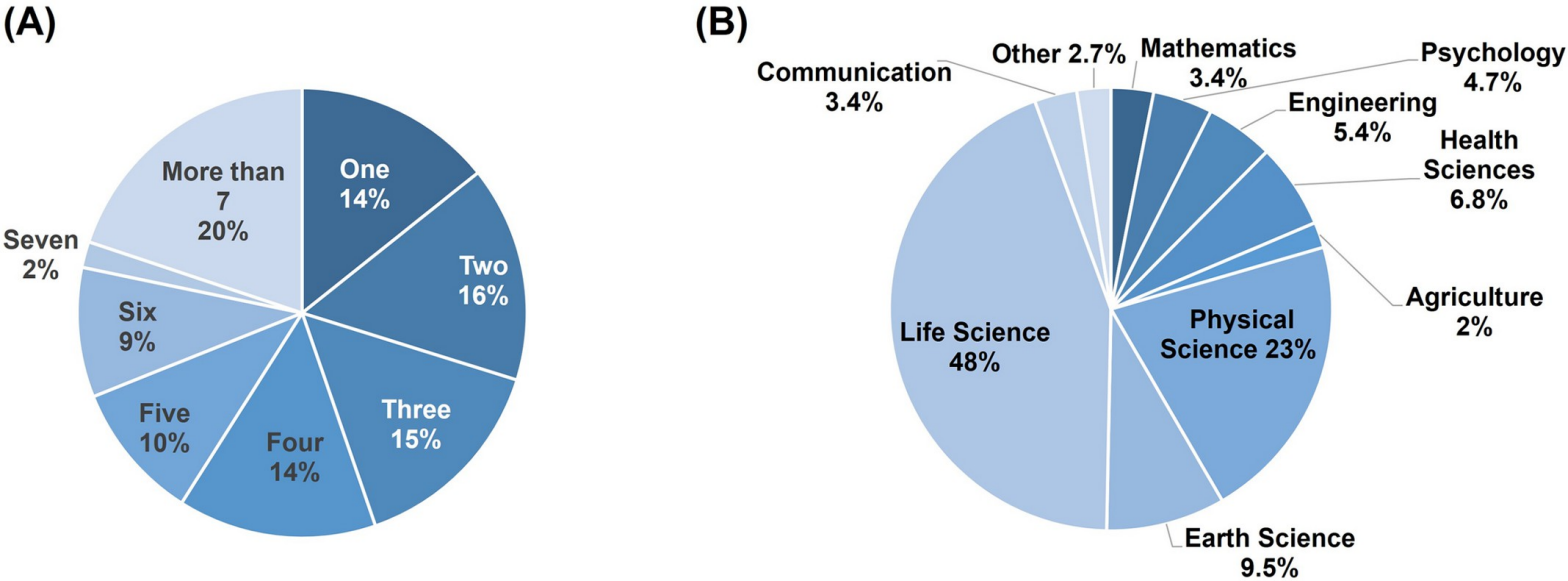
A full list of represented institutions can found in S1 File (S1 Table). A. Participants time completed in graduate school, in years, shown as percent responding. B. Participants subject-specific disciplines binned based on their branch of science following an existing organizational chart (https://www.mindmeister.com/1023614692/branches-of-science?fullscreen=1).

A full list of represented institutions can found in S1 File (S1 Table). Fig 1A shows participants’ time completed in graduate school at the time of the survey, indicating that there is roughly equal representation from participants at all levels of their graduate careers. Fig 1B shows participants were binned based on their field of study following an existing organizational chart (https://www.mindmeister.com/1023614692/branches-of-science?fullscreen=1).

### Performance/Competence and interest in science communication

A critical agency framework was adapted showing that feelings of performance and competence and prior interest in a STEM subject is a positive predictor of STEM identities [25, 26]. Specifically, performance and competency, measured as one construct, refer to an individual’s confidence levels in relation to their disciplinary community, which, for this study, would be the science communication community. In addition, interest in a particular subject has been shown to play a key role in a person’s choice of career, and for this study interest in communicating general science concepts with the public was measured.

Baseline measurements of performance/competence and interest in communicating general science concepts to the public were collected from participants. As this was a one-time survey, changes in these constructs over time will not be measured, but instead a general measurement of where participants fall on the performance/competence and interest spectrum as it relates to communicating general science concepts to the public. From these measurements, more will be learned about whether the participants exhibit performance/competence and identity as a science communicator and determine if performance/competence and interest measurements relate to any other identifying characteristics such as gender or teaching experience.

Five statements measuring performance/competence in communicating general science concepts to the public and 3 statements measuring interest in communicating general science concepts to the public were adapted from Godwin et al., [25]. "General science concepts" were defined as larger issues such as climate change, stem cell research, nuclear waste, drug development, gravitational waves, and other science concepts often included in scientific news reports. The statements are as follows:

Performance and competence

1. I am confident I can communicate general science concepts to the public;
2. I can accurately summarize and communicate general science concepts to the public;
3. I understand how to communicate general science concepts to the public;
4. I can overcome setbacks in communicating general science concepts to the public;
5. Others ask me for help in communicating general science concepts to the public.

Interest

1. I am interested in learning more about how to communicate general science concepts to the public;
2. Thinking about how to communicate general science concepts to the public excites my curiosity;
3. I enjoy learning about how to communicate general science concepts to the public.

Participants rated their agreement with each statement on a 5-point Likert scale from 0 (strongly disagree) to 4 (strongly agree). Scores for both performance/competence and interest were calculated as the average of responses to the 5 and 3 questions, respectively, with a higher score indicating higher levels of performance/competence and interest in communicate general science concepts to the public, which are suggestive of identity as a science communicator.

### Confirmatory Factor Analysis (CFA)

CFA was used to determine whether the performance/competence and identity questions adapted from Godwin (2016) were behaving the same way with this study’s student population as they were in the original study. Considering the small number of factors (two, performance/competence and interest) and the number of questions per factor (five for performance/competence and three for interest), the sample size of n = 161 is considered large enough to use for CFA [26, 27]. CFA was run using the R package lavaan [28]. It was assumed that the re-written prompts would represent two factors. A Comparative Fit Index (CFI) of 0.980, a robust RMSEA of 0.059, and a Standardized Root Mean Square Residual (SRMR) of 0.055 were found, all of which support that the adapted questions represent two factors as does the original scale [29].

### Chi-square test

A chi-square calculator was used to determine the association between two sets of categorical data, specifically training and engagement in science communication, using a 2x2 contingency table (www.socscistatistics.com). Any results that proved to be statistically significant were confirmed using R by running a Pearson’s Chi-squared test with Yates’ continuity correction. Cohen’s guidelines indicate that a 0.1 is considered a small effect, 0.3 is a medium effect and 0.5 is a large effect [30, 31].

### Mann Whitney

A Mann Whitney U calculator was used to determine if there was any difference in the performance/competence and interest for different sub-groups within samples, specifically looking at gender and TA/teaching experience (www.statskingdom.com). A nonparametric test was chosen for this analysis because the performance/competence and interest means were calculated using ordinal data (Likert scale) questions prior to the factor analysis. This calculator provided all of the information in Fig 6. U and p values were confirmed using the Wilcoxon rank sum test with continuity correction in R. Cohen’s guidelines indicate that a 0.1 is considered a small effect, 0.3 is a medium effect and 0.5 is a large effect [31, 32].

### Word clouds

**Fig 2:**
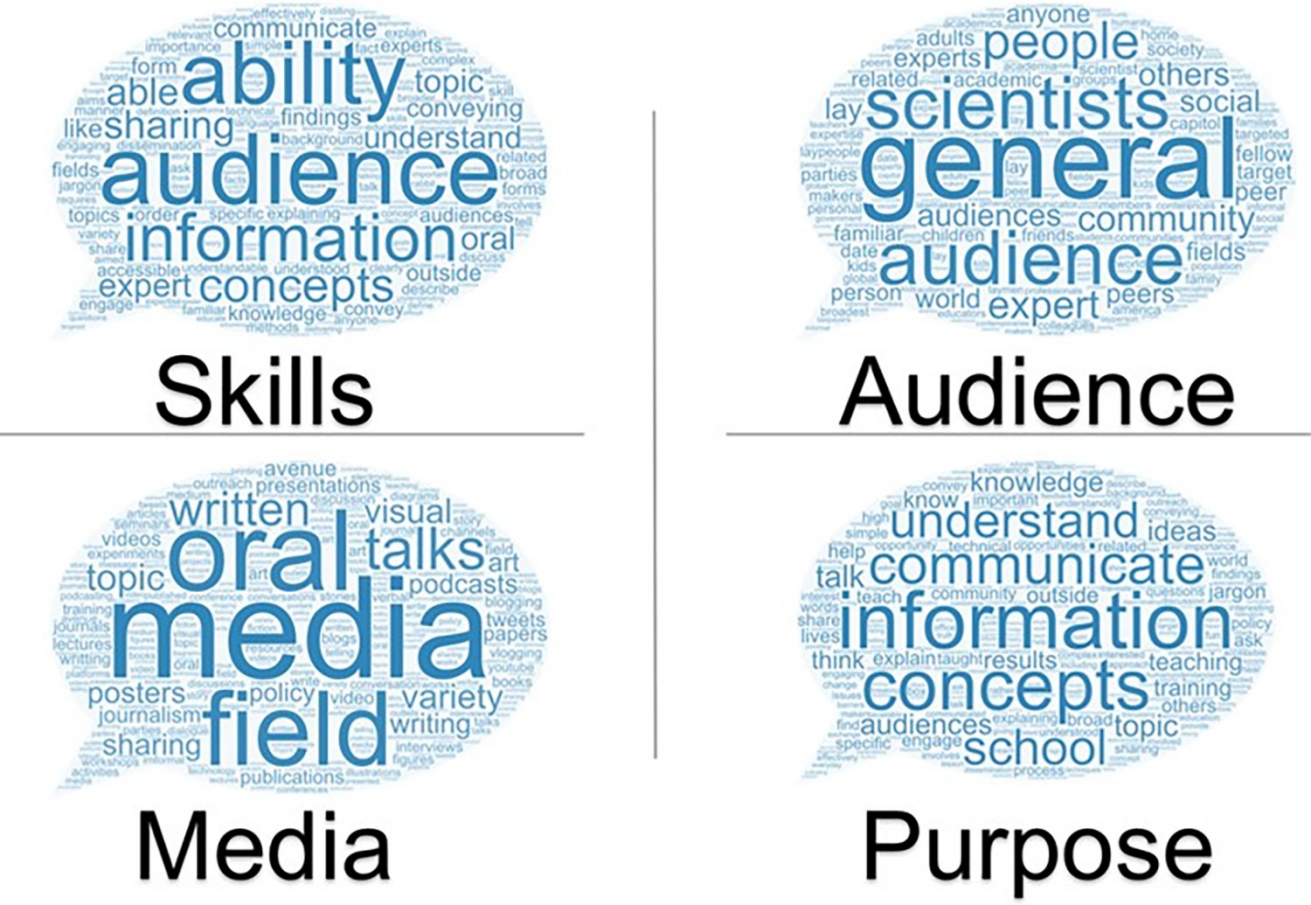
Word clouds highlight the complexity of participant definitions of science communication. The size of each of the words within the categorical word clouds correlates to its frequency in the coded responses. The words “science,” “scientific,” “communication,” and “communicating” have been removed in order to showcase more representative words.

Word clouds were used as a visual representation of participant responses to “How do you define science communication?” emphasizing the frequency of words used amongst participants. Word clouds were created based on the word frequency tables exported from the correlated Nvivo files. Word frequency tables included all words greater than three letters used in all of the coded questionnaire responses and the amount of times each word was used. Because the words science, scientific, communication, and communicating were used frequently among all participants and were not insightful for the purposes of this research, they were removed from the word tables. The word frequency tables were then inputted into WordArt.com to generate the word clouds (where the size of the words represent their frequency in the responses) included here (Fig 2). Raw data word clouds can be found in S1 File (S1 Fig).

## Results

### Participant demographics

A total of 161 responses from STEM graduate students in 29 U.S. states, 2 U.S. territories, and 11 countries were analyzed. A full list of represented Institutions is found in S1 File (S1 Table). The majority of participants reported that they are working towards a PhD degree (79%), 15% are working towards a master’s degree, 3% recently graduated with a PhD and 3% reported “other.” Participants are at varying stages of their graduate careers, with over 70% of participants enrolled in graduate school for at least three years or more (Fig 1A). A wide variety of disciplines are represented in the graduate student sample with the largest population pursuing degrees in life sciences (48%). Fig 1B includes discipline data from all responses. The gender ratio is 8 to 3, female to male, and the ethnic demographics are 71% white, 16% Asian, 11% Hispanic/Latino, 4% Multiracial, 1% Black or African American, 1% American Indian or Alaska Native, and 1% declined to respond.

### How do STEM graduate students define science communication?

Participants were asked: “In a few short sentences, how do you define science communication?” The qualitative responses received were complex and multifaceted. Inductive coding [23] was used to divide this data set into four main categories:

1. Skills STEM graduate students report that are needed to communicate science;
2. Intended audiences STEM graduate students expect to engage with;
3. Mediums through which STEM graduate students envision science communication; and
4. The purpose of science communication as perceived by STEM graduate students.

Once these main categories were established, inductive coding was used again to further define the data set found within each category. Results from inductive coding are shown in Tables 1–4 and are associated with category 1–4, respectively. To further understand the results of the inductive coding, four distinct word clouds corresponding each of the four categories were generated, highlighting the complexity of how STEM graduate students define science communication. (Fig 2).

**Tables 1–4:** Participant responses to the question “In a few short sentences, how do you define science communication?” To start, data was separated into four major categories. Table 1: **SKILLS,** skills STEM graduate students report being needed to communicate science. Table 2: **AUDIENCE,** intended audiences STEM graduate students expect to engage with. Table 3: **MEDIUM,** mediums through which STEM graduate students envision science communication. Table 4: **PURPOSE,** the purpose of science communication as perceived by STEM graduate students. Data within each of the four categories was further analyzed using inductive coding [23]. Tables 1–4 show the code (left column), a description of each code (middle column), and direct quotes from participants as examples (right column). Text from each direct quote that is relevant to each code is in bold and underlined text.

### Skills STEM graduate students report being needed to communicate science

A total of 73% of the participants who defined science communication using various skills included responses that fell under the code of “knowing your audience” (Table 1). It is unclear in the qualitative data whether participants listed this skill so often because it is a skill they have already acquired or if it is a skill that is somewhat “universal” in that it should always be considered when communicating. Participant responses also include “using clear language” (22% of participants), “knowing the science” (6% of participants), and “establishing relationships” (4% of participants). Only 2% of participants did not mention a skill in their response.

### Intended audiences STEM graduate students expect to engage with

The data indicates that STEM graduate students see two intended audiences for science communication: the general public (non-experts), mentioned in 50% of the responses, and other scientists (experts), mentioned in 2% of the responses. Thirty-four percent of participants defined science communication as involving both groups (Table 2). Eighteen percent of respondents did not mention an audience or described an audience in extremely general terms.

### Mediums through which STEM graduate students envision science communication

A majority of the respondents (76%) did not mention a medium within their definition of science communication, but for those who did, oral communication (15%) and written communication (13%) were mentioned most often. Social media was a distant third, with only 6% of respondents mentioning this in their definition.

### The purpose of science communication as perceived by STEM graduate students

A majority of respondents (78%) included the purpose of “sharing science with a general audience” as a way to increase appreciation of science, increase interest in science, and inspire confidence in science (Table 4). A much smaller amount of respondents (20%) described “sharing science with other scientists,” somewhat mirroring the data seen in Table 2 (50% consider their audience to be the general public, 2% consider their audience to be other scientists). Respondents mentioned other purposes of science communication include “informing policy” (16%); “educating others about science” (9%), which differs from “sharing science with a general audience” in that it deals with facts and misconceptions; and “encouraging the public to engage in science” (4%). Six percent of respondents did not mention a purpose.

### Science communication training at represented institutions

**Fig 3:**
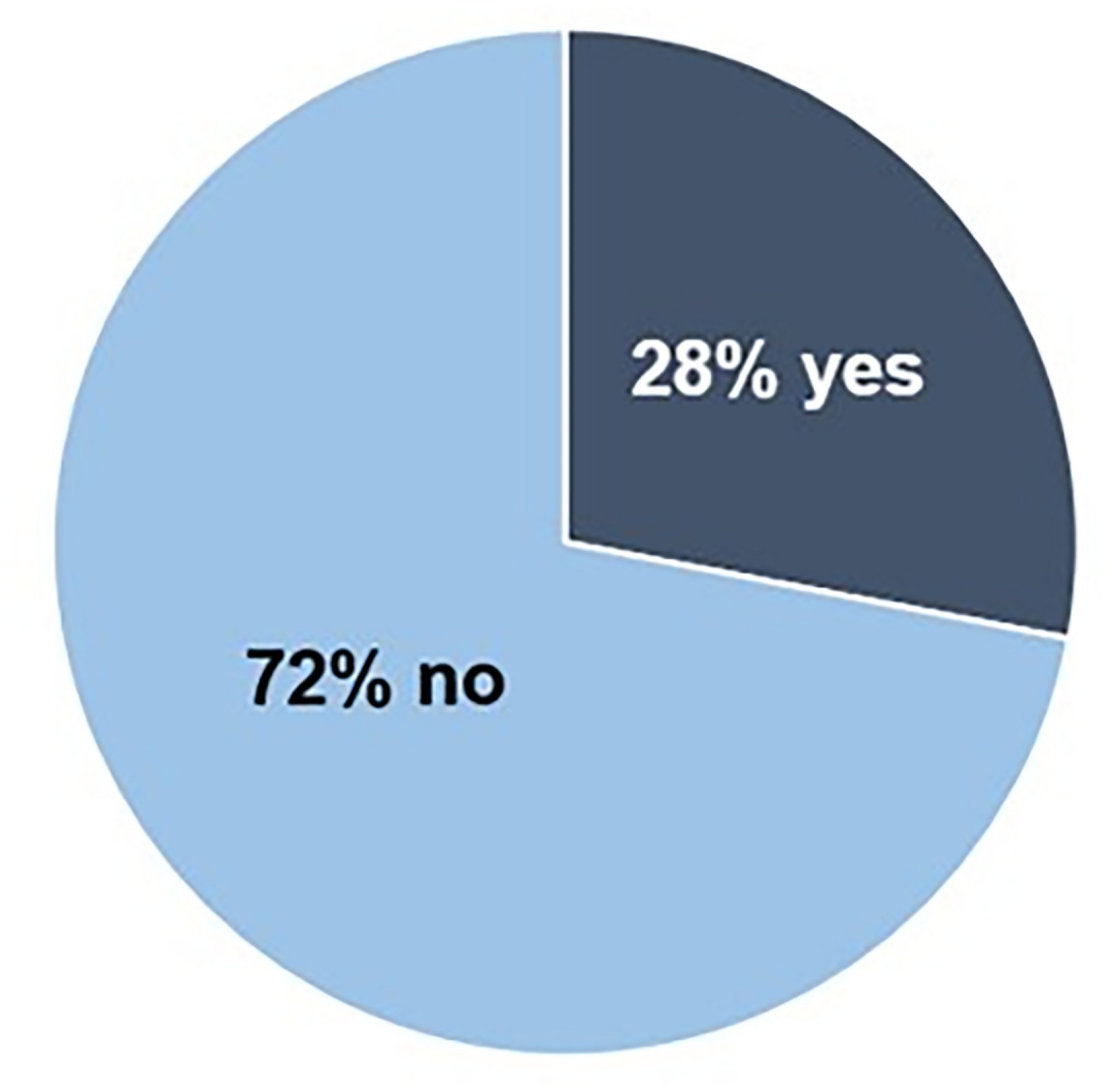
Participants report on whether their institution offers formal science communication training. Participant responses to the question “Did you have formal science communication training, for a public audience, at your graduate institution?” Data is shown as percent responding, with 72% responding no and 28% responding yes.

Participants were asked “Did you have formal science communication training, for a public audience, at your graduate institution?” Data is shown in Fig 3 as percent responding, with 72% responding no and 28% responding yes. It is possible that these institutions offer science communication training yet participants were unaware of available opportunities or were not able to engage in training.

**Fig 4:**
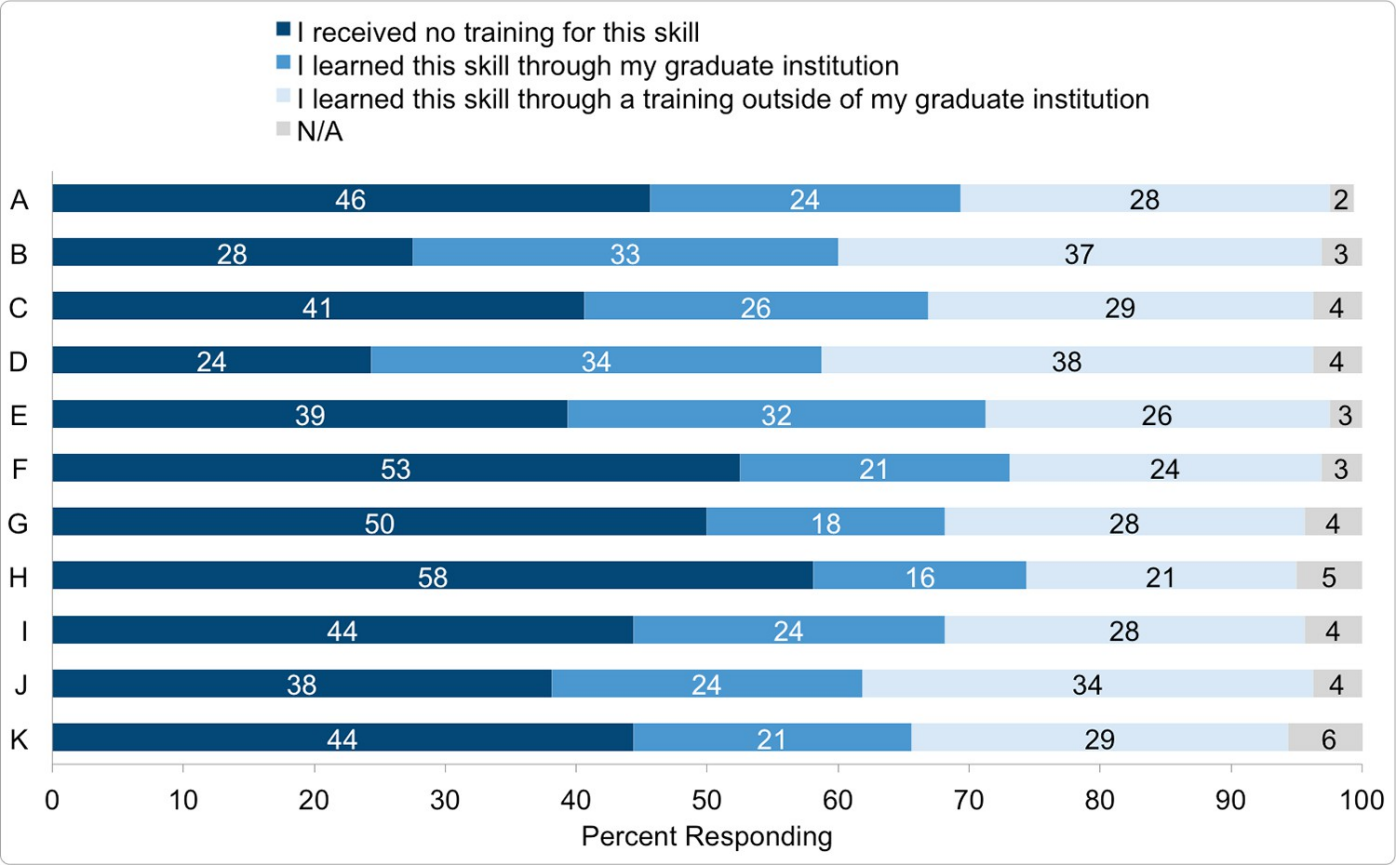
Participants previous training in specific science communication skills. Participant responses when asked where, if at all, they received training in 11 core skills in science communication, listed A-K [33]. Data are shown as percent responding.

Learning more about what science communication skills (if any) were being taught at graduate institutions was also of interest. For eleven of the core skills described by Mercer-Mapstone & Kuchel [33], participants were asked whether they learned this skill through their graduate institution, through a training outside of their graduate institution, or whether they received no training for this skill (Fig 4).

The majority of participants are reporting training with the exception of skills F, G, and H (skills F: use a suitable mode and platform to communicate with the target audience, G: consider the social, political, and cultural context of the scientific information, and H: understand the underlying theories leading to the development of science communication and why it is important are the only 3 skills where “no training” is above 50%).

The spread between the amounts of participants who received training in individual skills is large; e.g. 72% of the respondents receiving training in skill D, consider the levels of prior knowledge in my target audience, and only 37% of respondents received training in skill H, understand the underlying theories leading to the development of science communication and why it is important, suggesting that certain skills are focused on more than others.

With the exception of skill E, separate essential from non-essential factual content in a context that is relevant to the target audience, more science communication training takes place in outside graduate institutions, i.e. informal training, than within graduate institutions, i.e. formal training. As this was a multiple-choice question, we do not have additional information detailing what respondents meant when choosing “informal: outside graduate institution” training.

### What does science communication look like for today’s STEM graduate students?

**Fig 5:**
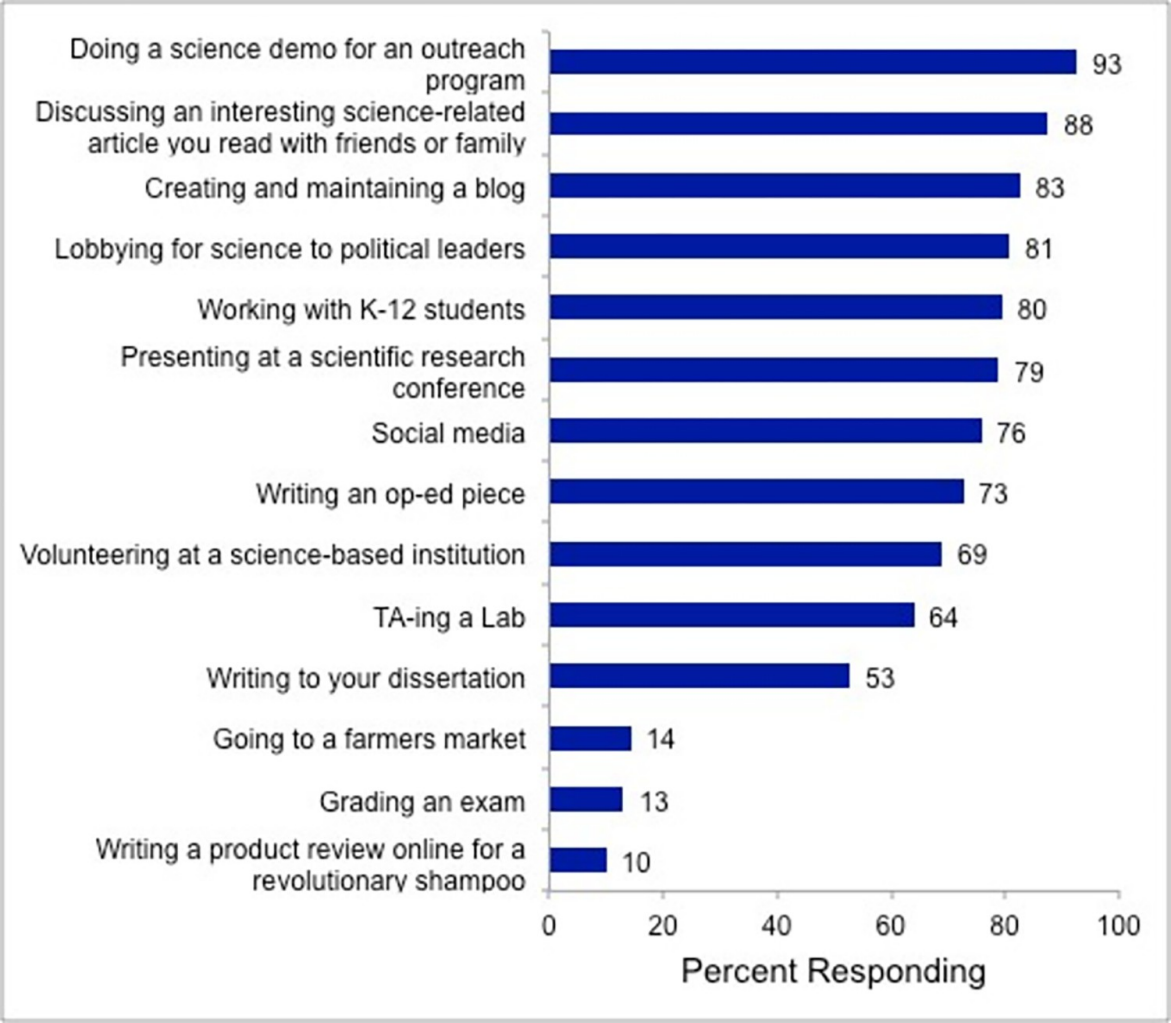
What do participants consider to be science communication? participants were asked “which of the following do you consider to fall within the category of science communication?” and directed to select all that apply. Data is shown as percent responding.

In order to understand what types of activities graduate students considered to be science communication, STEM graduate students were asked, “Which of the following do you consider to fall within the category of science communication?” Participants were able to choose from a list of activities, designed by the research team, shown in Fig 5, and were able to select as many as necessary. The top two selections being “Doing a science demo for an outreach program” (93%) and “Discussing science-related articles with a friend (88%).” These two selections, along with “Presenting at a scientific research conference” (79%) highlight oral communication among participants.

Science communication via online platforms and social media was prevalent in this data set (76%), but it was not the top choice, consistent with the data shown in Table 3. Participants seem more familiar with online platforms and social media; yet still tend to recognize more traditional forms of science communication.

“Lobbying for science to political leaders” was selected by 81% of participants, in contrast to the 16% who mention informing policy as a purpose of science communication in Table 4. This difference is likely due to the fact that respondents did not necessarily think about policy when defining science communication, but when asked about policy specifically in a multiple-choice question, they opted to include it.

A connection was seen to science education in this data set, specifically with the large number of participants choosing “Working with K-12 students” (80%) and “TA-ing a lab” (64%). There is also a connection to written communication, with “Creating and maintaining a blog” (83%), “Writing an op-ed piece” (73%), and “Writing your dissertation” (53%) all centered on strong writing skills.

### Communicating ‘general science’ -vs- communicating thesis research

We were also interested in learning more about what kind of science participants were communicating, specifically were they communicating "general science concepts" (defined as larger issues such as climate change, stem cell research, nuclear waste, drug development, gravitational waves, and other science concepts often included in scientific news reports; this definition was included in the question prompt) or were they communicating the details of their own thesis work. Roughly half of the sample (55%) reported engaging in general science concepts, just slightly higher than the 49% who reported communicating their thesis research, showing that participants were just as likely to communicate their own science as they were general science concepts (Table 7).

**Table 7:** Differences in audience types and qualitative responses between communicating general science concepts or thesis research as described by participants.

		General science concepts (Table 5)	Thesis research (Table 6)
Have you ever engaged in [science communication of general science concepts to the public?] or [your own thesis research] with the public?		55% yes	49% yes
Audience	K-12 audience	48%	17%
	Friends and Family	7%	19%
Barriers	Unaware of opportunities	15%	35%
	Overstepping expertise	27%	30%
	It is unnecessary	4%	13%
	Nerves	14%	4%
	Too busy	12%	4%
	Takes time away from the lab	7%	
	Thesis research is not transferrable to a general audience		11%

A series of qualitative questions were asked to further understand more about the experiences in science communication participants had and/or what was holding them back from engaging in science communication. Participants who responded”yes” to the question “do you engage in general science concepts?” were asked to “describe an example of how you have engaged in science communication related to a general science concept during your time in graduate school.” Inductive coding showed the data falling into two categories: 1) the types of audience respondents communicated with and 2) the types of medium respondents communicated using.

### Audience

Audiences participants engaged with included the general public (33%), elementary school students (19%), middle school students (17%), high school students (12%), and friends and family (7%). Other scientists were mentioned in only 6% of responses. Speaking to elected officials totaled 6% of responses.

### Medium

Mediums used for communicating general science concepts fell into three general themes of community outreach (54%), collaborations with schools (25%), and social media platforms (15%).

Participants who reported not engaging in general science concepts were asked, “What has stopped you from engaging in science communication related to a general science concept?” Inductive coding showed the data falling into six categories (Table 5). Half of respondents (51%) described being unaware of opportunities in their answer. Over one quarter of respondents (27%) felt they were overstepping their expertise, i.e. feeling unqualified to speak on research outside of their area of expertise. “Nerves,” (14%), being “too busy” with non-lab centered commitments (12%), unable to “take time away from the lab” (7%), and a feeling that science communication “is unnecessary” (4%) were additional reasons provided for why respondents had not engaged in science communication related to general science concepts.

Regarding participant’s thesis research, the same two questions were asked, starting with “Please describe an example of how you have engaged in science communication related to your thesis research during your time in graduate school.” Inductive coding showed similar results to what was seen with general science concepts, with a few exceptions. With regards to the audience the participants were interacting with, a decrease of respondents mentioning K-12 students as an audience (17%) for communicating thesis research was seen (48% of responses for general science concepts). This may be intuitive, as general science concepts are often more accessible and more applicable to real life.

A large shift was also seen in responses indicating that they discuss their science with friends and family (19%) compared to 7% of participants communicating general science. This may seem intuitive, as a commitment as intense as graduate school is highly likely to come up in conversation among friends and family. However, what was surprising was at the range of people participants were including: everyone from the dentist to the hairstylist to Tinder dates. A range of responses like this was not seen when asked about communicating general science concepts. Because of their unique nature, a variety of responses are listed in S1 File (S2 Table).

When participants were asked “what has stopped you from communicating your own thesis research to a non-scientific general audience?” Inductive coding showed data falling into six categories (Table 6). The number of responses relating to being “unaware of opportunities” was 35%, down considerably from the number of participants who described being unaware of opportunities for communicating general science (51%). Thirty percent of respondents described “overstepping expertise as a reason for not communicating thesis research, almost identical to the 27% seen with general science concepts. Thirteen percent of respondents indicated that communicating thesis research is unnecessary, higher than the number of participants who describe communicating general science as unnecessary (13% compared to 4%). Four percent of respondents described being afraid of the expectations of the public, being afraid to address controversial topics, and lack of confidence, which was coded together as “nerves.” Compared to general science concepts, the percentage of participants describing “nerves” as a barrier to science communication is lower (4% compared to 14%). The amount of participants citing lack of time to engage in science communication decreased (12% for general science concepts, 4% for thesis research). There were no responses describing “takes time away from the lab,” likely due to thesis research being fully connected to lab work. A new barrier, which emerged from this data set, was “Thesis research is not transferrable to a general audience” at 11%. Table 7 details similarities and differences in how respondents are communicating both general science concepts and their thesis research.

### Skills graduate students report being needed for communicating their thesis research

For participants answering yes to “Have you ever engaged in science communication of your own thesis research to the public?” we asked what they considered to be the most important skills they needed in order to do this (Table 8). “Knowing basic science communication techniques” was included in 65% of responses. “Knowing your audience” (48%), “knowing the science” (24%) and “engaging in two-way communication” (14%) were also included in participant responses.

**Table 8:** For participants who have communicated their thesis to the general public we asked: “what skills did you need?” the resulting codes are shown in the left hand column, with a description of each code in the center column, and example responses from participants in the right hand column. Text from each response that is directly relevant to each code is in bold and underlined text.

Code	Description of code	Example responses
Knowing basic science communication techniques (65% of respondents)	Participants describe reducing jargon, using visuals, using narratives and storytelling, and making the science accessible, exciting, and entertaining.	“Most important skills include b**eing able to connect with your audience**…. Also **breaking down scientific concepts with accessible language (i.e. keep jargon to a minimum** or explain them thoroughly), and **showing visuals** to explain data.” “**Explain base concepts** before I even think about talking about my project (Central dogma! What is cancer? How do we design cancer drugs?) without going into too much detail. **Use interesting visuals**--a general audience doesn’t want to have to interpret your data.”
Knowing your audience (48% of participants)	Participants describe skills such as being able to recognize the abilities and the knowledge level of their audience and being able to frame science in an interesting and engaging way.	“how to use very simple words to describe a technical concept and **how to read the public’s interest** in your thesis research during the conversation” “**Know your audience. Know your audience. Know your audience**.”
Knowing the science (24% of participants)	Participants describe the importance of knowing and understanding the science you are communicating.	“**Knowing the science** as best as possible” “**Understanding my own research better** has been the most important step. Communicating what I know is not so hard.”
Engaging in two-way communication (14% of participants)	Participants describe being able to admit they don’t know an answer and will work with the audience to figure it out.	“To be flexible and ready for anything. If you don’t know the answer to something they’re asking, **it’s okay to say I don’t know and potentially look up the answer together**!” “A non-scientific audience will always ask the unexpected questions. **It’s okay not to know something but it’s always useful to provide some direction for finding the answer**. Speaking with confidence creates a positive feedback loop.”

Finally, all participants were asked what additional training they think they would need in order to communicate their thesis research to the public (Table 9). Results were complex, with nine different codes emerging. One code, more opportunities, relates to codes seen previously in Tables 5 and 6 (unaware of opportunities). Certain concepts have also been seen before, such as jargon (Table 1). However, new and specific ideas for training opportunities that have not been present in previous data tables presented themselves. For example, turning research into a narrative and learning how to bring more storytelling to their thesis research (7%) and training in general public speaking (6%).

**Table 9:** We asked all participants “what additional training do you need to communicate your thesis research?” the resulting codes are shown in the left hand column, with a description of each code in the center column, and example responses from participants in the right hand column. Text from each response that is directly relevant to each code is in bold and underlined text.

Code	Description of code	Example responses
Including training in graduate school curricula (18% of respondents)	Participants describe their vision for science communication training as a part of graduate school.	"I think this would be a good idea to **incorporate in the college curriculum**. I think it would be a good idea to be able to discuss your thesis to a general audience.” "It would be great if **a short course or even a few-day workshop was required for all graduate students**."
More opportunities to practice (17% of respondents)	Participants discuss needing opportunities to practice rather than more training.	"I’m not sure if training is the correct goal. **Ultimately it takes practice**, consistency, and a bit of guidance (much like getting your PhD) to become an expert science communicator. I think the best thing people can do is start with their friends and family and **take every opportunity** to speak with the public so they can refine those skills." "**More opportunities to present**, experiences in formats besides a standard talk (written, podcast, etc.)"
None (13% of respondents)	Participants are confident in their ability to communicate their thesis research.	"I think I’m good. I gotta finish my thesis now. Lol! **I’ve had a really great training**, but I had to go look for it. None of the opportunities were advertised within my program." "**I believe I have sufficient experience and training to communicate my thesis research to any audience**—therefore, I’m not sure I can identify any more than I would need to communicate it to a non-scientific audience."
Help making the science accessible (8% of respondents)	Participants describe needing guidance with simplifying jargon, coming up with real world connections, and how to excite and engage an audience.	"I don’t think it would be tough **but I can learn more engaging and interaction technique**s." "How to make **convos interactive not me lecturing them**."
More practice writing (8% of respondents)	Participants describe needing more training in written communication skills.	"I could always use more training on **how to better write for a non scientific audience**. I am very interested in getting training on how to be concise and pithy to use social media as a communication tool." "**Training focusing on writing techniques** instead of oral science communication"
Turning my research into a narrative (7% of respondents)	Participants describe needing to learn how to connect storytelling to their thesis research.	"**Storytelling training. Learning how to tell a good story** might help me deliver my work in a way that is more accessible and engaging." "Not training so much as **a narrative to my research. Right now it is not a story**, and it doesn’t lend itself to the general audience."
Public speaking training (6% of respondents)	Participants describe needing training in general public speaking skills.	"How to make presentations and **how to improve our projection when speaking****."** "**Better presentation skills with powerpoints and speaking**, as well as a more technical understanding of grammar."
Visual media training (6% of respondents)	Participants describe needing training in creating videos, pictures, power point slides, and graphics.	"a good platform that gets to a large audience of general public. **help developing material (simple figures, cartoons****)** and direction etc." "I’d like to learn **how to make videos and infographics**."
How to read an audience (6% of respondents)	Participants describe needing training in evaluating what a general audience already knows and misconceptions general audiences might have.	"**How to better judge an audience’s interest and knowledge level** would be helpful." "I do not know what the public does and does not know. Additionally, **the public has a lot of pre-conceived notions about science that I do not know**. Before I communicate what I do, I need to understand what their notions are so I can understand where they are coming from."
How to translate jargon (5% of respondents)	Participants describe needing training in making complex scientific concepts and language easier to understand.	"Training on **how to translate scientific jargon into everyday language** would be extremely helpful." "Basic training about **how to explain complicated concepts in simple ways**, and how to identify what information you should explain, and what kind of information is too much."

### Interest and performance/competence as it relates to science communication

**Fig 6:**
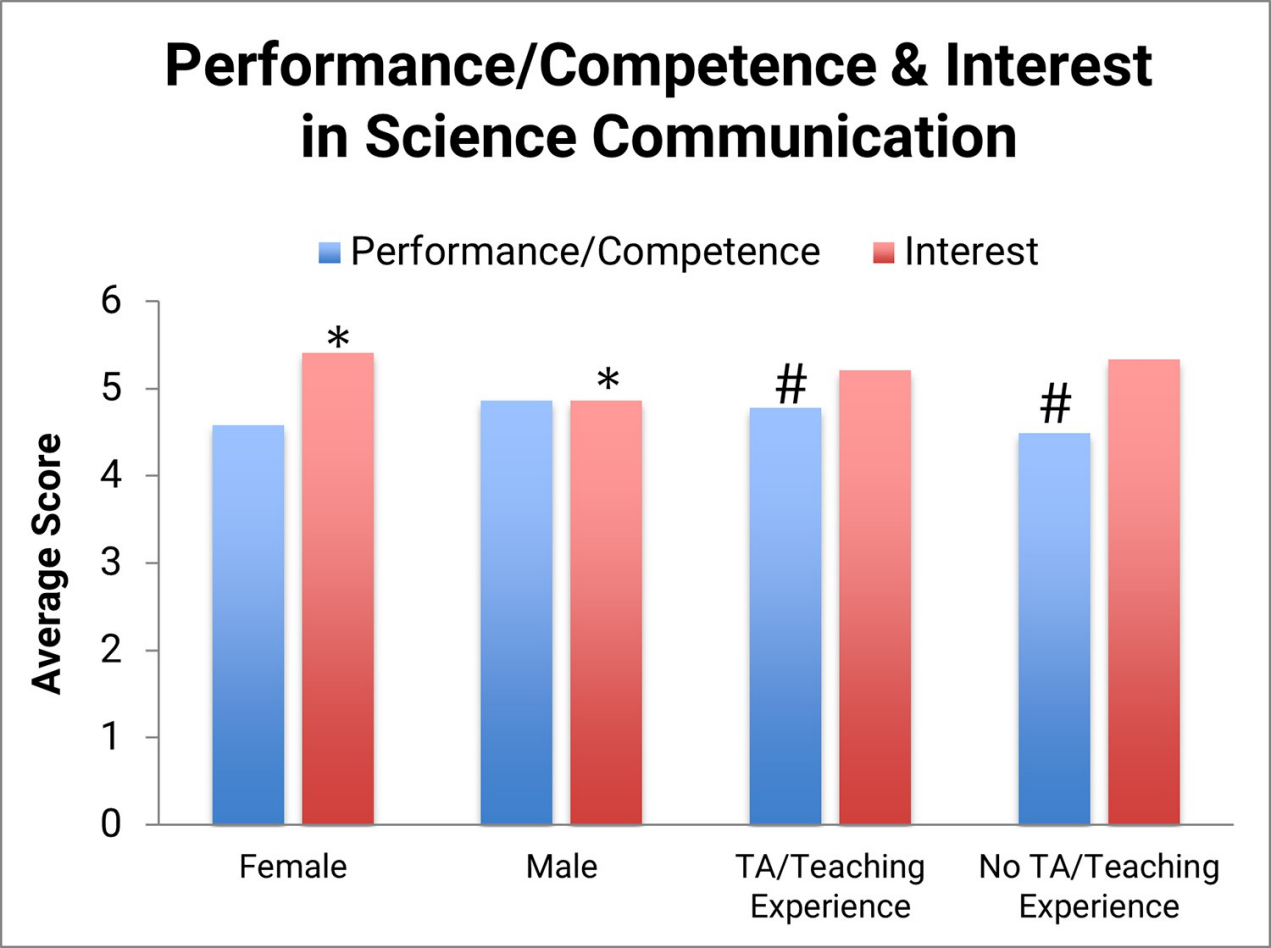
Participant performance/competence and interest and in science communication. Further statistical tests were done to examine relationships between interest and competence/performance in science communication, gender, and previous teaching experience. Bars with asterisks and hashtags indicate a statistically significant difference between two groups, gender and teaching experience, respectively. A corresponding data chart is found in S1 File (S3 Table).

Critical agency frameworks previously developed in STEM education were adapted as a way to measure participant performance/competence and interest relating to science communication (see methods). Because the questionnaire was only given once, these data serve solely as a starting point for new lines of inquiry into STEM graduate students perceptions of science communication. The performance/competence level was found to be 4.6 (out of 6) and the interest level was found to be 5.2 (out of 6), which indicate a high starting level for both (likely the result of a self-selected population who completed a science communication questionnaire for no incentive) (Fig 6). These data suggest that STEM graduate students believe they are able to engage in science communication and that they have a strong interest in science communication, although the self-selection bias of participants likely affects this result.

These measures were also analyzed in the context of other data points. Specifically, were there any differences in performance/competence and/or interest based on participant’s gender and/or prior teaching experience? While there was no statistically significant gender difference between the competence scores, there was a statistically significant difference between female and male interest in science communication (*p* < 0.05) with a moderate effect size where *r* = 0.22, showing that women in this study expressed a higher interest in science communication (Fig 6, S1 File (S3 Table)). We also confirmed a statistically significant difference between the means for performance/competence (but not interest) between participants who have had prior TA/teaching experience and those who have not with a moderate effect size where *r* = 0.20. In summary, women in this study showed a higher interest in science communication, and those participants with previous teaching experience showed a higher mean performance/competence score than those without prior teaching experience (Fig 6, S1 File (S3 Table)). These results can serve as a basis for future studies and can be used to guide new training programs.

## Discussion

The ability to communicate effectively is an important skill for scientists regardless of career path; therefore, science communication training should be an integral component of graduate-level coursework. In this study, we expand the knowledge base on how current STEM graduate students define science communication, how they practice and engage in science communication, and how they envision their future as science communication practitioners. STEM graduate students’ lived experiences as future science communicators are characterized through data presented here.

STEM graduate students have a very complex and multi-faceted view of science communication (Tables 1–4). This is encouraging, as participants in this study, who represent the future of science communication, view science communication in line with our initial definition: scientists sharing their work inside their community and science with non-experts [1]. Collectively, participants report science communication encompassing various skills, audiences, platforms, and purposes. Perhaps most inspiring about this data is the emphasis of two-way communication tactics, such as knowing your audience, establishing relationships, and tailoring your messages based on your audiences’ needs (Tables 1 and 8). Two-way communication is essential for successful science communication between scientists and the public, and thus the importance of emphasizing it more during science communication training is critical [34]. Participants in this study already seem to embrace this concept, suggesting that they are primed and ready for their future roles as science communicators.

We learn a little more about how graduate students see themselves as science communicators, specifically with regards to the general public. The performance/competence data from Fig 6, which refer to an individual’s confidence levels in relation to their disciplinary community, which, for this study, is the science communication community, are relatively high, suggesting that participants in this study have confidence in their ability to communicate with the public. Roughly half of the participant sample (55%) reported engaging in general science concepts with the general public, which is just slightly higher than the 49% who reported communicating their thesis research, suggesting that participants are equally likely to share their thesis work as they are general science. While we do see some instances of imposter syndrome, for example 27% reported feeling unqualified to speak on research outside of their area of expertise (Table 5), these seem to be the minority of responses. Collectively, participants seem to be comfortable communicating with the public. While we did not specifically ask whether students consider themselves to be expert or novice communicators, or where they would place themselves on this spectrum, these would be interesting topics for future research studies on how graduate students explicitly identify as science communicators. Expert–novice comparisons are valuable research tools as they provide practical insights into how to aid novices in developing more expert-like skills and learning more about what the expert-novice science communication spectrum looks like would be useful in developing best practices for science communication training.

Seventy two percent of participants responded “no” to the question, “Did you have formal science communication training, for a public audience, at your graduate institution?” This means either that the opportunity to did not exist at participants’ graduate institutions or the opportunity existed but was unknown to the participant. Coupled with the fact that students received no incentives to complete this survey, this result becomes even more disappointing, as this population likely self-selected from students with an understanding of and interest in science communication who may have been acutely aware of and/or actively seeking out offerings through their institution.

A separate dataset in this study investigated whether graduate students received training in 11 core science communication skills (Fig 4). While the majority of participants reported training in 8 out of 11 skills, these data show that more science communication training takes place outside graduate institutions than within graduate institutions for 10 of the 11 core skills.

Taken together, these datasets are sobering, because whether or not science communication trainings are formally offered through graduate institutions is, at its core, an equity issue. When offered as a part of graduate school, science communication training can be built into students’ course load, accepted and respected by advisors, and paid for with tuition credits or waivers. When students are instead compelled to leave their institutions to seek these trainings, trainings become available only to those students who have the time, support, and financial means, as well as those who are aware of existing opportunities, which these data show to be the biggest barrier to science communication training (Table 7).

Formally including science communication training and opportunities into graduate curricula would remove not only the barriers listed above, but alleviate additional barriers found in these data including, the view that science communication is unnecessary, students being too busy for science communication, and students being unable to take time away from the lab (Table 7). Two additional barriers listed in the dataset, overstepping expertise and nerves, both can be overcome with practice and exposure, further reinforcing the need to make science communication opportunities a part of the official graduate school curricula. Additionally, when asked what additional training students need, the number one response was to include training in graduate student curricula (Table 9). While changing graduate school curricula is not trivial, these data point to a single solution that would remove several different barriers. Lifting barriers is a step closer to a more equitable environment.

Data presented in this study also provides the graduate student perspective in the larger conversation larger conversation regarding what should be key characteristics of science communication trainings, and can be used as a blueprint for designing future science communication trainings that are formally offered by graduate institutions. When asked what additional training participants would need, results were again multifaceted (Table 9). Requests for training in two-way communication are prevalent, as are requests for general training in public speaking, both of which suggest future science communicators who are truly interested in connecting with their audiences. Perhaps most inspiring are the requests for learning how to turn research into a narrative and how to bring more storytelling to science communication, an aspect of science communication that is currently being promoted and encouraged in research studies and trainings [35].

STEM graduate students will serve as science ambassadors throughout their careers; thus it is imperative that we hear from graduate students about their science communication training experiences, how they define science communication, how they have engaged in the process of science communication, and how they envision the future of science communication training. Graduate school administrators and all other stakeholders should consider these data when creating science communication training to ensure equitable and impactful opportunities for all.

## Supporting information

S1 Questionnaire Grad student attitudes towards science communication. (PDF)

S1 File (DOCX)

S1 Data Survey data. (XLSX)
