# Supplementary material for: How do STEM graduate students perceive science communication? Understanding science communication perceptions of future scientists
Source: PLoS One. 2022 Oct 3;17(10):e0274840. doi: 10.1371/journal.pone.0274840 (PMC9529114; doi:10.1371/journal.pone.0274840)
Supplement: S1 Questionnaire — (PDF) [file pone.0274840.s001.pdf]

# Grad student attitudes towards science communication

Start of Block: Default Question Block

Q1 **ADULT CONSENT TO PARTICIPATE IN A RESEARCH STUDY** Graduate Student Communication Training Survey **PURPOSE OF THE STUDY.** You are being asked to be in a research study. The purpose of this research is to document experiences relating to science communication at the graduate and expert level. If you choose to participate, your participation will involve either completing a short survey with the option for a follow up interview or participating in an expert science communicator interview. **NUMBER OF STUDY PARTICIPANTS.** If you decide to be in this study, you will be one of around 500 people in this research study. **DURATION OF THE STUDY.** Your participation will require you to complete an online survey (15 minutes) with the option for a follow up interview (60 minutes) or participate in an interview as a science communication expert (60 minutes). **PROCEDURES** If you agree to be in the study, we will ask you to do the following things: 1. complete an online survey (15 minutes) with the option for a follow up interview (60 minutes), OR; 2. participate in an interview as a science communication expert (60 minutes). **RISKS AND/OR DISCOMFORTS.** There are no known risks associated with this study. **BENEFITS.** The following benefits may be associated with your participation in this study: Improve the quality of graduate science communication training Enhance our understanding of what makes a successful science communicator **ALTERNATIVES.** There are no known alternatives available to you other than not taking part in this study. **CONFIDENTIALITY** The records of this study will be kept private and will be protected to the fullest extent provided by law. In any sort of report we might publish, we will not include any information that will make it possible to identify a subject. Research records will be stored securely and only the researcher team will have access to the records. However, your records may be reviewed for audit purposes by authorized University or other agents who will be bound by the same provisions of confidentiality. **COMPENSATION & COSTS** You will receive no compensation for your participation. You will not be responsible for any costs to participate in this study. **RIGHT TO DECLINE OR WITHDRAW** Your participation in this study is voluntary. You are free to participate in the study or withdraw your consent at any time during the study. Your withdrawal or lack of participation will not affect any benefits to which you are otherwise entitled. The investigator reserves the right to remove you without your consent at such time that they feel it is in the best interest. **RESEARCHER CONTACT INFORMATION** If you have any questions about the purpose, procedures, or any other issues relating to this research study you may contact Dr. Melissa McCartney at OE 230, 305-348-7165, mmccartn@fiu.edu. **IRB CONTACT INFORMATION** If you would like to talk with someone about your rights of being a subject in this research study or about ethical issues with this research study, you may contact

the FIU Office of Research Integrity by phone at 305-348-2494 or by email at ori@fiu.edu.

**PARTICIPANT AGREEMENT**

○ I agree to participate in this research study (1)

Page Break

Q2 How many years have you been in graduate school?

- ○ less than 1 (1)
- ○ 1 (10)
- ○ 2 (2)
- ○ 3 (3)
- ○ 4 (4)
- ○ 5 (5)
- ○ 6 (6)
- ○ 7 (7)
- ○ more than 7 (8)
- ○ other (13) ________________________________________________

Page Break

Q3 What general field of science are you in?

________________________________________________________________

Page Break

Q4 Where is your current university located?  This is to ensure we have a sample of responses from across the world.

- ○ US - based  (4)
- ○ non US - based  (5)

Page Break

*Display This Question:*

*If Where is your current university located? This is to ensure we have a sample of responses from ac... = US - based*

Q5 Please enter the state your institution is located in

________________________________________________________________

Page Break

*Display This Question:*

*If Where is your current university located? This is to ensure we have a sample of responses from ac... = non US - based*

Q6 Please enter the country your institution is located in

__________________________________________________________________

Page Break

Q7 Please provide the name of your institution.  This will be used to categorize our respondent pool using Carnegie classifications.

________________________________________________________________

Page Break

Q8
What is the highest degree you have earned to date?

- ○ undergraduate degree (1)
- ○ masters degree (3)
- ○ other (4) ________________________________________________

Q9
What type of degree are you currently pursuing?

- ○ masters degree (1)
- ○ PhD (2)
- ○ I am not currently pursuing a degree (4)
- ○ other (3) ________________________________________________

Page Break

Q10 Are you Hispanic or Latino?

- yes (1)
- no (3)

---

*Display This Question:*

*If Are you Hispanic or Latino? = yes*

Q11 Which of the following best describes your Hispanic origin or descent?

- Mexican American or Chicano (1)
- Puerto Rican (2)
- Cuban (3)
- Other Hispanic (4)

---

*Display This Question:*

*If Are you Hispanic or Latino? = no*

Q12 What is your race?  Please select all that apply.

- ☐ White  (4)
- ☐ Black or African American  (5)
- ☐ American Indian or Alaska Native  (6)
- ☐ Asian  (7)
- ☐ Native Hawaiian or Pacific Islander  (8)
- ☐ Multiracial  (9)
- ☐ Prefer not to answer  (10)

Page Break

Q13 To which gender do you most identify?

- ◯ Woman (1)
- ◯ Man (2)
- ◯ Transgender Woman (3)
- ◯ Transgender Man (4)
- ◯ Nonbinary/nonconforming (5)
- ◯ Other (7)
- ◯ Prefer not to answer (6)

Page Break

Q14 In a few short sentences, how do you define science communication?

(Answers should be 150 words or less).

Page Break

Q15 Do you have any teaching or TA experience? **Please refer only to your time in graduate school.**

○ yes (1)

○ no (2)

Page Break

*Display This Question:*

*If Do you have any teaching or TA experience?  Please refer only to your time in graduate school.  = yes*

*

Q16 In a few short sentences, please describe your teaching experience **during your graduate school training.**

(Answers should be 150 words or less).

________________________________________________________________

________________________________________________________________

________________________________________________________________

________________________________________________________________

________________________________________________________________

Page Break

*Display This Question:*

*If Do you have any teaching or TA experience?  Please refer only to your time in graduate school.  = yes*

*

Q17
What is your approach when communicating science to your students? Is it similar/different to when you communicate science to the public?

Please refer only to your time in graduate school.

(Answers should be 150 words or less).

________________________________________________________________

________________________________________________________________

________________________________________________________________

________________________________________________________________

________________________________________________________________

Page Break

Q18
For this question, "general science concepts" is defined as larger issues such as climate change, stem cell research, nuclear waste, drug development, gravitational waves, and other science concepts often included in scientific news reports.

To what extent do you agree or disagree with the following statements?

When engaging in science communication with the general public (a non-scientific general audience),

| | Strongly disagree (1) | Disagree (2) | Slightly disagree (3) | Slightly agree (4) | Agree (7) | Strongly Agree (5) | N/A (6) |
|---|---|---|---|---|---|---|---|
| I am confident I can communicate general science concepts to the public. (1) | ○ | ○ | ○ | ○ | ○ | ○ | ○ |
| I can accurately summarize and communicate general science concepts to the public. (2) | ○ | ○ | ○ | ○ | ○ | ○ | ○ |
| I understand how to communicate general science concepts to the public. (3) | ○ | ○ | ○ | ○ | ○ | ○ | ○ |
| I can overcome setbacks in communicating general science concepts to the public. (4) | ○ | ○ | ○ | ○ | ○ | ○ | ○ |

| | | | | | | | |
|---|---|---|---|---|---|---|---|
| Others ask me for help in communicating general science concepts to the public. (5) | ○ | ○ | ○ | ○ | ○ | ○ | ○ |
| I am interested in learning more about how to communicate general science concepts to the public. (6) | ○ | ○ | ○ | ○ | ○ | ○ | ○ |
| Thinking about how to communicate general science concepts to the public excites my curiosity. (7) | ○ | ○ | ○ | ○ | ○ | ○ | ○ |
| I enjoy learning about how to communicate general science concepts to the public. (8) | ○ | ○ | ○ | ○ | ○ | ○ | ○ |

Page Break

Q19 Did you have formal science communication training, for a public audience, at your graduate institution?

- yes (1)
- no (2)

Page Break

Q20
For this question, "general science concepts" is defined as larger issues such as climate change, stem cell research, nuclear waste, drug development, gravitational waves, and other science concepts often included in scientific news reports.

Have you ever engaged in science communication of "general science concepts" to the public?  Please refer only to your time in graduate school.

- ○ Yes  (1)
- ○ No  (2)

Page Break

*Display This Question:*

*If For this question, "general science concepts" is defined as larger issues such as climate change,... = Yes*

Q21 What type of science communication was this?

- ◯ Formal (1)
- ◯ Informal (2)
- ◯ Both (5)
- ◯ other (4) ________________________________________________

Page Break

*Display This Question:*

*If For this question, "general science concepts" is defined as larger issues such as climate change,... = Yes*

Q22 What type of audience did you present to?  Please check all that apply.

- ▢ elementary students  (6)
- ▢ middle school students  (7)
- ▢ high school students  (8)
- ▢ undergraduate students  (12)
- ▢ general public  (1)
- ▢ mixed group  (4)
- ▢ virtual  (13)
- ▢ other scientists (not in my field)  (14)
- ▢ other  (5) ________________________________________________

Page Break

*Display This Question:*

*If For this question, "general science concepts" is defined as larger issues such as climate change,... = Yes*

Q23 Please describe an example of how you have engaged in science communication related to a general science concept.

Please refer only to your time in graduate school.

(Answers should be 150 words or less).

________________________________________________________________

________________________________________________________________

________________________________________________________________

________________________________________________________________

________________________________________________________________

Page Break

*Display This Question:*

*If For this question, "general science concepts" is defined as larger issues such as climate change,... = No*

*

Q24 What has stopped you from engaging in science communication related to a general science concept?

Please refer only to your time in graduate school.

(Answers should be 150 words or less).

________________________________________________________________

________________________________________________________________

________________________________________________________________

________________________________________________________________

________________________________________________________________

Page Break

Q25 Have you ever engaged in science communication of your own thesis research to a non-scientific general audience?

Please refer only to your time in graduate school.

○ Yes (1)

○ No (2)

Page Break

*Display This Question:*

*If Have you ever engaged in science communication of your own thesis research to a non-scientific ge... = No*

*

Q26 What has stopped you from communicating your own thesis research to a non-scientific general audience?  Please feel free to describe positive and/or negative experiences.

Please refer only to your time in graduate school.

(Answers should be 150 words or less).

________________________________________________________________

________________________________________________________________

________________________________________________________________

________________________________________________________________

________________________________________________________________

Page Break

*Display This Question:*

*If Have you ever engaged in science communication of your own thesis research to a non-scientific ge... = Yes*

*

Q27 Please describe how you communicated your own thesis research to the public (a non-scientific general audience).

Please refer only to your time in graduate school.

(Answers should be 150 words or less).

________________________________________________________________

________________________________________________________________

________________________________________________________________

________________________________________________________________

________________________________________________________________

Page Break

*Display This Question:*

*If Have you ever engaged in science communication of your own thesis research to a non-scientific ge... = Yes*

*

Q28 What would you say are the most important skills you have learned in order to communicate your own thesis research to the public (a non-scientific general audience)?

Please refer only to your time in graduate school.

(Answers should be 150 words or less).

________________________________________________________________

________________________________________________________________

________________________________________________________________

________________________________________________________________

________________________________________________________________

Page Break

*Display This Question:*

*If Have you ever engaged in science communication of your own thesis research to a non-scientific ge... = Yes*

Q29 When reflecting on this communication experience, how effective do you think you were?

- ◯ not effective (1)
- ◯ slightly effective (2)
- ◯ effective (3)
- ◯ very effective (4)
- ◯ N/A (5)

Page Break

*Display This Question:*

*If Have you ever engaged in science communication of your own thesis research to a non-scientific ge... = Yes*

Q30 Where did you receive your science communication training? Please refer only to your time in graduate school.

◯ At my graduate institution (1)

◯ I found training opportunities outside of my graduate institution (for example: a workshop at a conference, a webinar) (2)

◯ I never had any training (3)

◯ other (4) ________________________________________________

Page Break

Q31 What additional training do you think you need in order to communicate your own thesis research to the public (a non-scientific general audience)?

(Answers should be 150 words or less).

________________________________________________________________

________________________________________________________________

________________________________________________________________

________________________________________________________________

________________________________________________________________

Page Break

Q32 Please consider the following core skills for effective science communication.

Did you gain any of these skills directly through training provided by your graduate institution?

Did you gain any of these skills through participating in trainings outside of your graduate institution?

Did you not receive training for any of these skills?

| | I learned this skill through my graduate institution (1) | I learned this skill through a training outside of my graduate institution (2) | I received no training for this skill (3) | N/A (4) |
|---|---|---|---|---|
| identify and understand a suitable target audience (1) | o | o | o | o |
| use language that is appropriate for my target audience (2) | o | o | o | o |
| identify the purpose and intended outcome of a science communication project (3) | o | o | o | o |
| consider the levels of prior knowledge in my target audience (4) | o | o | o | o |
| separate essential from non-essential factual content in a context that is relevant to the target audience (5) | o | o | o | o |

| | | | | |
|---|---|---|---|---|
| use a suitable mode and platform to communicate with the target audience (6) | ○ | ○ | ○ | ○ |
| consider the social, political, and cultural context of the scientific information (7) | ○ | ○ | ○ | ○ |
| understand the underlying theories leading to the development of science communication and why it is important (9) | ○ | ○ | ○ | ○ |
| promote audience engagement with the science (10) | ○ | ○ | ○ | ○ |
| use the tools of storytelling and narrative (11) | ○ | ○ | ○ | ○ |
| encourage a two-way dialogue with the audience (12) | ○ | ○ | ○ | ○ |

Page Break

Q33 Which of the following do you consider to fall within the category of science communication?
Choose all that apply.

- ▢ Doing a science demo for an outreach program (1)
- ▢ Writing a product review online for a revolutionary shampoo (2)
- ▢ TA-ing a Lab (3)
- ▢ Grading an exam (4)
- ▢ Writing to your dissertation (5)
- ▢ Lobbying for science to political leaders (6)
- ▢ Presenting at a scientific research conference (7)
- ▢ Going to a farmers market (8)
- ▢ Writing an op-ed piece (9)
- ▢ Creating and maintaining a blog (10)
- ▢ Social media (11)
- ▢ Volunteering at a science-based institution (12)
- ▢ Working with K-12 students (15)
- ▢ Discussing an interesting science-related article you read with friends or family (16)
- ▢ other (13) ________________________________________________

Page Break

Q34 In general, what are your career plans after graduate school?

________________________________________________________________

Page Break

Q35 How did you hear about this survey?

________________________________________________________________

Page Break

Q36 Would you be interested in being contacted for an interview after completing this survey? If so, please provide your email address in the space below.

- Yes (1) ________________________________________________
- No (3)

End of Block: Default Question Block

---
