# Supplementary material for: How do STEM graduate students perceive science communication? Understanding science communication perceptions of future scientists
Source: PLoS One. 2022 Oct 3;17(10):e0274840. doi: 10.1371/journal.pone.0274840 (PMC9529114; doi:10.1371/journal.pone.0274840)
Supplement: S1 File — (DOCX) [file pone.0274840.s002.docx]

How do STEM Graduate students perceive science communication? Understanding science communication perceptions of future scientists.

Tessy S. Ritchie ^1^, Dione L. Rossiter ^2^, Hannah Bruce Opris^3,4^, Idarabasi Evangel Akpan^3,4^, Simone Oliphant^3,4^, and Melissa McCartney^3,4*^

^1^Department of Chemistry & Life Science, United States Military Academy, West Point NY

^2^Science at Cal, University of California, Berkeley, Berkeley, CA

^3^STEM Transformation Institute, Florida International University, Miami FL

^4^Department of Biological Sciences, Florida International University, Miami FL

***Correspondence:**Corresponding Author
[mmccartn@fiu.edu](mailto:mmccartn@fiu.edu)

**This PDF includes:**

S1 Figure

S1-S3 Tables

**S1 Table: Institutions represented in this study.** A complete list of Institutions represented in this study and the number of participants linked to each institution.

| **Institution** | **# of respondents** |
| --- | --- |
| Arizona State University | 2 |
| Azusa Pacific University | 1 |
| Bowling Green State University | 1 |
| California Institute of Technology | 7 |
| California State University Fullerton | 2 |
| Cornell University | 1 |
| Duke Kunshan University | 1 |
| Florida International University | 13 |
| GCER (Global Centre for Environmental Remediation) | 1 |
| Georgetown University | 1 |
| Harvard University | 1 |
| Illinois State University | 1 |
| Indiana University | 2 |
| Iowa State University | 4 |
| ISCTE | 1 |
| Johns Hopkins University | 1 |
| liverpool john moores university (astrophysics research institute) | 1 |
| Mississippi state university | 1 |
| National Taiwan Normal University | 1 |
| North Carolina State University | 2 |
| Northeastern University | 1 |
| Northwestern University | 6 |
| The Ohio State University | 3 |
| Oregon Health & Science University | 1 |
| Oxford university | 1 |
| Royal Holloway, University of London | 1 |
| San Diego State University | 3 |
| Scuola Internazionale di Studi Superiori Avanzati (SISSA) di Trieste | 1 |
| SISSA | 1 |
| Stanford | 1 |
| Stem Cell biology institution | 1 |
| Texas A&M University | 2 |
| The Australian National University | 1 |
| The University of Kansas | 1 |
| The University of Sheffield | 1 |
| The University of Texas at Austin | 1 |
| The University of Western Australia | 1 |
| Tufts University | 3 |
| UC Berkeley | 3 |
| UC Irvine | 5 |
| Univ of Illinois Urbana Champaign | 1 |
| University of Houston | 1 |
| University of Aberdeen | 1 |
| University of Adelaide | 1 |
| University of Alabama at Birmingham | 2 |
| University of Arizona | 1 |
| University of California Los Angeles | 6 |
| University of California, Davis | 5 |
| University of California, Santa Barbara | 2 |
| University of Colorado - Boulder | 2 |
| University of Florida | 2 |
| University of Georgia | 3 |
| University of Guam | 1 |
| University of Idaho | 1 |
| University of Illinois at Chicago | 1 |
| University of Maryland, College Park | 5 |
| University of Massachusetts Amherst | 1 |
| University of Massachusetts Boston | 1 |
| University of Massachusetts Dartmouth | 2 |
| University of Michigan | 2 |
| University of Minnesota | 3 |
| University of Mississippi | 3 |
| University of Nebraska-Lincoln | 1 |
| University of Newcastle | 1 |
| University of North Carolina at Chapel Hill | 1 |
| University of North Texas Health Science Center | 1 |
| university of Pennsylvania | 1 |
| University of Pittsburgh | 4 |
| University of Potsdam | 1 |
| University of Pretoria | 1 |
| University of Puerto Rico | 1 |
| University of Rochester | 1 |
| University of South Australia | 1 |
| University of South Florida | 1 |
| University of Tennessee Knoxville | 2 |
| University of Utah | 1 |
| University of Virginia | 1 |
| University of Washington | 6 |
| University of Washington - Seattle | 1 |
| University of Washington Tacoma | 1 |
| University of Wisconsin-Madison | 1 |
| Vanderbilt University | 1 |
| Virginia Commonwealth University (School of Medicine) | 1 |
| Wageningen University & Research | 1 |

**S1 Figure**: **Word cloud data.** A single word cloud was created using all of the reviewed word lists regarding the question to summarize all the responses to the question as a whole.

| **All words** | **Without skew words** | **Final words** |
| --- | --- | --- |
| Audience | | |
| 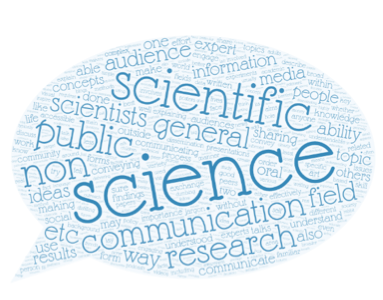 | 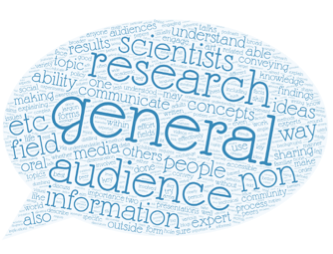 | 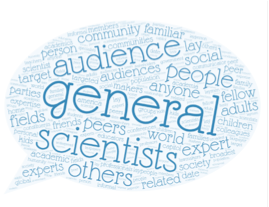 |
| Purpose | | |
| 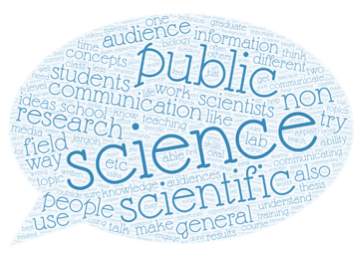 | 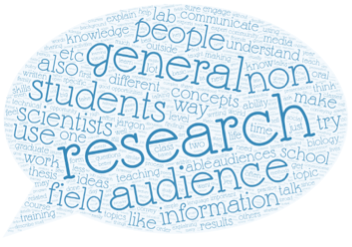 | 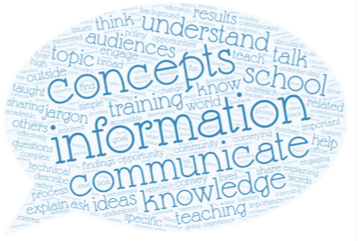 |
| Mediums | | |
| 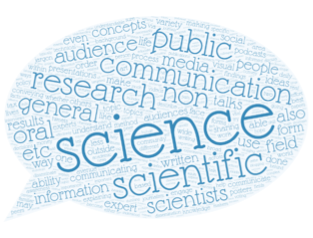 | 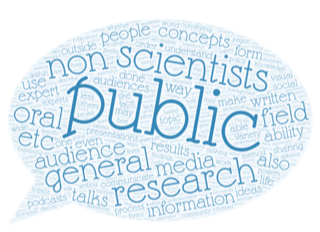 | 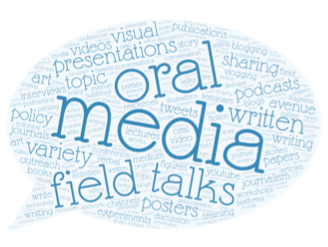 |

| Skills | | |
| --- | --- | --- |
| 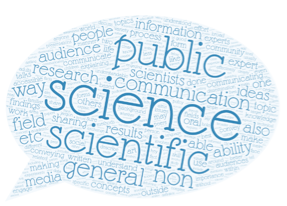 | 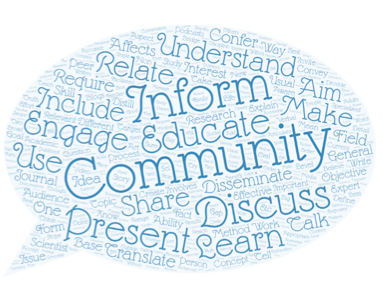 | 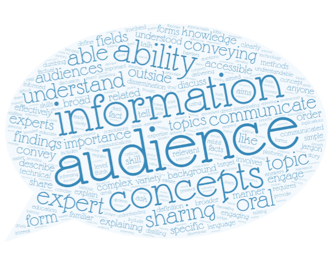 |
| Summary | | |
| 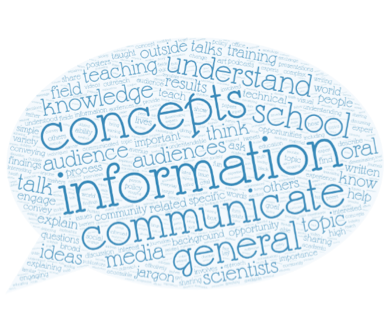 | | |

**S2 Table**. **Participant responses to the question “Have you ever engaged in science communication of your own thesis research to a non-scientific general audience?”**. Data shown are the responses falling under the theme of “communicating with my family and friends.”

| **Example responses** |
| --- |
| “I often communicate my research to friends and family. Most often **Uber and Lyft** drivers are the most excited to hear about my research because saying "I'm a scientist" is a job description that they don't hear every day.”  “Usually to a **group of friends**. Very informal. Would love to do a 3mt one day.”  “Casual conversations with friends, family, and **residents in my town**.”  “I have given my "elevator pitch" to anyone who has asked; from family members to a **dental hygienist.**”  “I have never communicated my thesis research to the public in a formal setting, but I often talk about it with people I meet. When people ask me about my job and I say I study astronomy, they are often interested to hear more and I'm happy to talk about it. I have talked about my thesis research with family members, **Uber drivers, hairstylists, friends of friends, people sitting next to me on planes, people I met at jury duty**, and so on.”  “Talking to family, friends, and **Tinder dates.**”  “Lots of **people from my hometown** have very little science background, so when I speak with them, I bring up general concepts first like Alzheimer's disease and air pollution.” |

**S3 Table.** **The corresponding data chart for Figure 6.** Participant performance/competence and interest and in science communication. Further statistical tests were done to examine relationships between interest and competence/performance in science communication, gender, and previous teaching experience.

|  |  | **N** | **Mean** | **Median** | **U-Value** | **z-score** | ***p*** | **Stat. Sig?*** |
| --- | --- | --- | --- | --- | --- | --- | --- | --- |
| Competence | Female | 106 | 4.58 | 4.6 | 2522.5 | -1.77 | 0.0761 | No |
|  | Male | 40 | 4.86 | 5 | 1717.5 |  |  |  |
|  | Total | 146 |  |  |  |  |  |  |
| Interest | Female | 106 | 5.41 | 5.67 | 1540.5 | 2.62 | **0.0088** | Yes |
|  | Male | 40 | 4.86 | 5.17 | 2699.5 |  |  |  |
|  | Total | 146 |  |  |  |  |  |  |
|  | | | | | | | | |
| Competence | TA/Teaching Experience | 96 | 4.78 | 4.8 | 1941.5 | 2.4 | **0.016** | Yes |
|  | No TA/Teaching Experience | 53 | 4.49 | 4.6 | 3146.5 |  |  |  |
|  | Total | 149 |  |  |  |  |  |  |
| Interest | TA/Teaching Experience | 96 | 5.21 | 5.67 | 2573.5 | -0.12 | 0.904 | No |
|  | No TA/Teaching Experience | 53 | 5.33 | 5.33 | 2514.5 |  |  |  |
|  | Total | 149 |  |  |  |  |  |  |
| * Significance level is 0.05 | |  |  |  |  |  |  |  |
